# Distribution Shifts of *Acanthaster solaris* Under Climate Change and the Impact on Coral Reef Habitats

**DOI:** 10.3390/ani15060858

**Published:** 2025-03-17

**Authors:** Shangke Su, Jinquan Liu, Bin Chen, Wei Wang, Jiaguang Xiao, Yuan Li, Jianguo Du, Jianhua Kang, Wenjia Hu, Junpeng Zhang

**Affiliations:** 1 Key Laboratory of Marine Ecological Conservation and Restoration, Third Institute of Oceanography, Ministry of Natural Resources, Xiamen 361005, China; sushangke@tio.org.cn (S.S.); liujinquan@tio.org.cn (J.L.); chenbin@tio.org.cn (B.C.); dujianguo@tio.org.cn (J.D.); kangjianhua@tio.org.cn (J.K.); 2 Laboratory of Marine Biodiversity Research, Third Institute of Oceanography, Ministry of Natural Resources, Xiamen 361005, China; wangwei@tio.org.cn (W.W.); xiaojiaguang@tio.org.cn (J.X.); liyuan@tio.org.cn (Y.L.); 3 APEC Marine Sustainable Development Center, Xiamen 361005, China; 4 Ocean Dynamics Laboratory, Third Institute of Oceanography, Ministry of Natural Resources, Xiamen 361005, China

**Keywords:** crown-of-thorns starfish, climate change, coral reef, species distribution model, distribution change

## Abstract

Pacific crown-of-thorns starfish (*Acanthaster solaris*) outbreaks pose a significant threat to coral reef ecosystems, with climate change potentially exacerbating their distribution and impact. However, there remains only a small number of predictive studies on how climate change drives changes in the distribution patterns of *A. solaris*, and relevant assessments of the impact of these changes on coral reef areas are lacking. To address this issue, this study investigated potential changes in the distribution of *A. solaris* under climate change and its impact on *Acropora* coral habitats. Using a novel two-step framework, we integrated both abiotic and biological (*Acropora* distribution) predictors into species distribution modeling to project future shifts in *A. solaris* habitats. We created the first reliable set of current and future global distribution maps for *A. solaris* using a comprehensive dataset and machine learning approach. The results showed significant distribution shifts under three climate change scenarios (SSP1-2.6, SSP2-4.5, and SSP5-8.5), with expanded ranges under all scenarios, and the greatest expansion occurring near 10° S. Asymmetry in the latitudinal shifts in habitat boundaries suggests that the Southern Hemisphere may face a more severe expansion of *A. solaris*. Regions previously unsuitable for *A. solaris*, such as parts of New Zealand, might experience new invasions. Additionally, our findings highlight the potential increase in predatory pressure on coral reefs under SSP2-4.5 and SSP5-8.5 scenarios, particularly in the Western Coral Triangle and Northeast Australian Shelf, where an overlap between *A. solaris* and *Acropora* habitats is significant. This study provides critical insights into the ecological dynamics of *A. solaris* in the context of climate change, and the results have important implications for coral reef management. These findings highlight the need for targeted conservation efforts and the development of mitigation strategies to protect coral reefs from the growing threat posed by *A. solaris*.

## Simple Summary

*Acanthaster solaris* outbreaks are a major threat to coral reefs, and climate change may worsen their spread. Despite this, there have been few studies predicting how climate change affects their distribution and the impact on coral habitats. This study aimed to predict how *A. solaris* might shift its range under climate change and how it could impact coral reefs, particularly *Acropora* corals. Using a two-step species distribution modeling approach, the study created the first global maps of current and future *A. solaris* habitats. The results revealed that the starfish’s range could expand, especially in the Southern Hemisphere, with some areas like New Zealand potentially seeing new invasions. The findings also indicated that regions with abundant *Acropora* corals could face increased predatory pressure, particularly under high-emission climate scenarios. This research is important for coral reef management and highlights the urgent need for targeted conservation efforts to protect these ecosystems from the growing threat of *A. solaris*.

## 1. Introduction

Pacific crown-of-thorns starfish (*Acanthaster solaris*) belong to the class Asteroidea of the phylum Echinodermata and are known for their ability to damage coral reef ecosystems [1,2]. *A. solaris* is a large starfish that feeds on coral and is one of the most notorious coral-eating invertebrates in the coral reefs of the Indo-Pacific region [3]. During periodic population outbreaks, the local density of *A. solaris* can increase from very low (<1 starfish per hectare) to extremely high (>1000 starfish per hectare) [4]. Coral reefs are characterized by their high productivity and biodiversity [5], supporting various marine species and playing a crucial role in maintaining the health and balance of ocean ecosystems. Outbreaks of *A. solaris* can lead to changes in local coral cover, composition, community structure, and coral-associated organisms, thereby affecting the entire ecosystem [6]. Despite the global decline in coral reefs in recent years due to a range of stressors such as ocean warming, ocean acidification, and tropical cyclones [7], predation by crown-of-thorns starfish (COTS) remains one of the greatest threats to coral reefs [8]. Statistics from over 200 coral reefs worldwide show that between 1985 and 2012, up to 42% of coral cover loss was attributed to CoTS outbreaks [9]. On the Great Barrier Reef, between 2016 and 2020, predation by *A. solaris* accounted for 11% of annual coral mortality, second only to the impacts of bleaching and cyclones [10].

*A. solaris* is one of the five corallivorous *Acanthaster* species primarily distributed in the western Pacific Ocean and eastern Indian Ocean, with the Great Barrier Reef of Australia being its most well-known habitat [11]. Moreover, it is also found in the coral reef ecosystems of Japan, the Philippines, Indonesia, China, the Maldives, and Sri Lanka [8,12]. It exhibits a high level of specificity towards certain coral species, such as those of the *Acropora* genus. Owing to their rapid growth and high nutritional content, *Acropora* corals are among the preferred food sources for *A. solaris* [13]. Although juvenile COTS are herbivorous, adult COTS primarily feed on coral tissue [14]. In areas with limited coral availability or under specific environmental conditions, COTS may occasionally consume soft corals, sponges, and mollusks [15,16]. However, these alternative food sources make up only a small portion of their diet, making it difficult for COTS to find viable food sources outside of coral reef habitats. Most other coral-eating organisms can only cause localized damage or tissue loss in corals, whereas adult *A. solaris* can kill all corals or even relatively large coral colonies. Each adult *A. solaris* can consume up to 10 m^2^ of hard coral per year [17]. In the Great Barrier Reef, outbreaks of *A. solaris* have had a far greater impact on corals than the combined losses caused by tropical storms and coral bleaching [18,19]. This region has experienced four large-scale outbreaks of *A. solaris* since the 1960s [6,20], with clear indications of a fifth outbreak. Coral reefs in French Polynesia, Indonesia, Okinawa, the Xisha Islands, and the Spratly Islands have also been severely affected [21,22,23,24,25]. Coral reef ecosystems serve as habitats and breeding grounds for various marine fish species, and the destruction of corals affects fish habitats and reproduction. Consequently, *A. solaris* outbreaks can lead to significant economic losses for local aquaculture and marine fisheries [6,26,27]. The predatory impact of *A. solaris* can also cause the loss of coral reef landscapes, which in turn affects the marine tourism industry, especially in tourist destinations that focus on activities such as diving and snorkeling [28,29].

Climate change may alter the distribution patterns of *A. solaris*. Studies have shown that rising water temperatures can promote the growth of *A. solaris* populations and even drive them to migrate to areas that are usually cooler [30]. Moreover, ocean acidification may reduce the resistance of certain coral species, providing more readily available food resources for *A. solaris,* facilitating its spread within coral reef ecosystems [13,31]. In damaged coral reef areas, the population of *A. solaris* may increase rapidly [6,11], thereby altering its distribution. However, studies of *A. solaris* are relatively scarce, possibly because, until recently, the genus *Acanthaster* (*Acanthaster* spp.) was considered a widely distributed single species (*Acanthaster planci*); it was only with the aid of DNA barcoding that it was confirmed that the genus contains at least five species [8]. Evidence suggests that owing to climate change, the *A. solaris* habitat is expanding into areas where it was previously absent. For example, observations in Japan show that its northernmost distribution, previously recorded at Amami Oshima in 1945, extended northward to Miyake Island and Goto Island in 2015 (with an average northward shift of approximately 500 km) [32]. Additionally, new outbreaks have occurred at locations where *A. solaris* has not been previously reported. For instance, *A. solaris* outbreaks were first reported in Brunei in 2010 and Nha Trang Bay, Vietnam, in 2017 [33,34,35]. These changes are believed to be closely related to rising sea temperatures, increased nutrient availability (leading to higher primary productivity), and coral reef bleaching. However, there remains only a small number of predictive studies on how climate change drives changes in the distribution patterns of *A. solaris*, and relevant assessments of the impact of these changes on coral reef areas are lacking.

To address this issue, this study aimed to investigate the distributional changes of *A. solaris* under climate change and assess the impact on coral reef (*Acropora*) habitats. The objectives of this study were as follows: (1) to predict the distribution of *A. solaris* under three climate change scenarios for the year 2100 using machine learning-based species distribution models (SDMs) and identify the environmental constraints on its distribution; (2) to evaluate the spatial pattern changes of *A. solaris* under different climate scenarios; (3) to analyze the potential impact of these changes on coral reefs (*Acropora*). This research provides essential data for the ecosystem risk management of *A. solaris* and lays a foundation for helping coral reefs to cope with predator challenges posed by climate change.

## 2. Materials and Methods

### 2.1. Study Area

**Figure 1:**
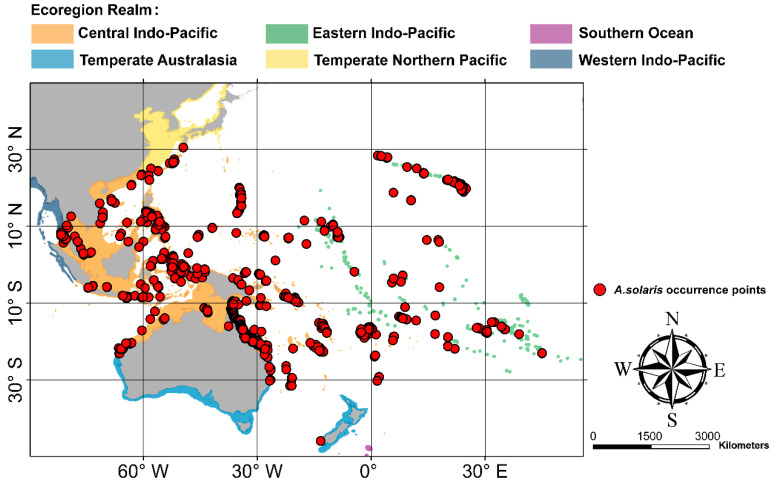
Map of the study area and occurrence points of *A. solaris*.

Based on the biogeographic distribution of the different species of *Acanthaster* [8], we defined the research area as the known distribution area of *A. solaris* and the surrounding waters, with a longitudinal range of 98.28° E–134.97° W and a latitudinal range of 45.91° S–30.46° N (Figure 1). According to the World Marine Ecoregions (WME) classification, this area encompasses 6 realms and 28 provinces, with a total area of 10,595,034.12 km^2^.

### 2.2. Abiotic and Biotic Variables

The application of species distribution modeling (SDM) to simulate the distribution of *A. solaris* requires relevant marine environmental variables as predictors. Fluctuations in temperature and salinity significantly affect the development and distribution of *A. solaris* [36,37,38,39]. Current velocity can influence the success of larval diffusion and fertilization [40,41,42]. Nutrient concentrations, pH, bathymetry, turbidity, and other factors also affect the distribution of benthic organisms, such as starfish [38,43,44,45,46,47]. Consequently, 32 environmental variables were selected as alternative abiotic factors for the model (Table S1). Furthermore, unlike some benthic species that depend on physical substrates such as mud or sand, *A. solaris* is a specialized coral-associated species [28,48,49], with *Acropora* coral species forming its primary habitat. Therefore, coral habitats are likely to play a key role in shaping the geographic distribution of *A. solaris*. Therefore, in addition to abiotic factors, we incorporated the distribution of *Acropora* corals as a biological factor and the distance from coral reefs as an environmental variable in the SDM.

The abiotic variable data were sourced from the Bio-ORACLE v3.0 and GEBCO datasets [50,51]. These data are widely used in macroecological research [46,52]. From these, we obtained benthic marine environmental variables for the present decade (2010–2020) and for the end of the 21st century (2090–2100) under the SSP1-2.6, SSP2-4.5, and SSP5-8.5 climate scenarios [50]. SSP5-8.5 represented the worst-case scenario, SSP2-4.5, the baseline scenario, and SSP1-2.6, the sustainable development scenario, respectively [53]. Owing to a lack of future predictions with low uncertainty for water depth and diffuse attenuation, these were treated as static variables that remained unchanged over time. The original data had a spatial resolution of 2.5 arcmin, which was adjusted to 5 arcmin using the resampling tool in ArcGIS 10.2. We quantified the correlation between the predictor variables using Pearson’s correlation coefficient (r) and excluded variables with high collinearity (|r| > 0.7) to minimize the impact of multicollinearity on model fitting (Figure S1) [54]. Specifically, an iterative elimination process was employed to address collinearity. In each iteration, the variable with the highest average absolute correlation within the group was identified and removed. This process was repeated until no highly correlated variables exceeding the predefined threshold remained in the dataset. By iterating this method, we ensured that the final set of variables included only those that were sufficiently independent, minimizing the impact of multicollinearity on the model. Based on the collinearity results and ecological relevance, we selected 12 abiotic variables as model inputs (Table 1). For biological variables, we first simulated the distribution of *Acropora* corals under different climate scenarios using SDMs and then calculated the distance from the coral reefs based on the Euclidean distance method. The spatial resolution used in this process was also 5 arcmin.

### 2.3. Species Distribution Data

Historically, species of the genus *Acanthaster* (*Acanthaster* spp.) have often been regarded as a widely distributed single species (*A. planci*), and as a result, different *Acanthaster* species have been recorded as *A. planci* in global databases [8]. Based on the distribution framework of various *A. solaris* species proposed by Uthicke et al., 2024 [8], we reconstructed a distribution dataset specifically for *A. solaris*. We first collected all occurrence records of *A. planci* from four databases: the Global Biodiversity Information Facility (https://www.gbif.org/, accessed on 14 July 2024), the Ocean Biodiversity Information System (https://obis.org/, accessed on 14 July 2024), iNaturalist (https://www.inaturalist.org/, accessed on 14 July 2024), and the Atlas of Living Australia (https://www.ala.org.au/, accessed on 14 July 2024). These records were then spatially filtered according to the *A. solaris* distribution ecoregions as defined by Uthicke et al. (2024) [8], resulting in 11,483 occurrence points for *A. solaris*. In addition, to provide biological variables for the *Acropora* genus, we obtained 289,440 global occurrence records for *Acropora* species from the aforementioned databases.

We employed a series of data-cleaning procedures to ensure the reliability of the species’ occurrence data used in the modeling process. Initially, we used the “spocc” R package version 1.2.0 [55] to remove records with invalid or duplicate coordinates. Subsequently, to reduce the potential sampling bias in model fitting, we retained only one occurrence record per 5 arcmin grid cells (as the resolution for environmental variables) [56,57]. After filtering, we obtained 945 occurrence points for *A. solaris* and 6642 occurrence points for *Acropora* in the modeling process.

### 2.4. SDM Training and Prediction

To reduce the overexpression of unsuitable environmental conditions within the region and minimize computational load, we used the defined coastal and shelf ecosystems from the World Ocean Biogeographic Atlas [58] as spatial filters, thereby identifying an appropriate modeling area [59]. The modeling process was carried out in two steps. First, we modeled the habitat suitability for *Acropora* corals using 12 abiotic variables to obtain current and future reef distributions. In the *A. solaris* modeling process, we applied the dependency of *A. solaris* on *Acropora* coral habitats as a constraint, incorporating the distance from coral reefs as a biological variable to simulate the current and future distributions of *A. solaris*.

In the selection of modeling algorithms, we used MaxEnt version 3.4.1 in combination with marine abiotic environmental variables to simulate species habitat suitability [60,61]. MaxEnt is a classical machine learning algorithm that performs well in predicting species distributions when only presence data are available and is the most widely used modeling algorithm in marine research [62]. The selection of background points plays a crucial role in the modeling process [63]. With reference to the limitations of species dispersal capacity in model evaluation [64,65], we created a 2000 km buffer around the distribution datasets of *Acropora* genus and *Acanthaster* spp. and selected 10,000 random points within this buffer as background data [66]. We optimized the regularization parameters and feature classes of the MaxEnt model using the “ENMeval” R package version 2.0.3. By combining different regularization multipliers and feature class combinations, we constructed 30 candidate MaxEnt models for each species and evaluated their predictive performance using 5-fold spatial cross-validation. The optimal model was determined based on the 10% omission rate, area under the receiver operating characteristic curve (AUC), and true skill statistics (TSS) [67,68,69]. The best model was applied to predict the species habitat suitability under current and future climate conditions, and the continuous suitability layers were converted into binary distribution maps (ranging from 0 to 1) using equal training sensitivity and specificity logistic thresholds [60,61]. To ensure reliable results, we independently modeled the habitat suitability for each species following the ODMAP protocol provided in Table S2 [70].

### 2.5. Assessing the Impact of A. solaris Distribution on Coral Reef Habitats

We evaluated the effects of *A. solaris* distribution on coral reef habitats from two perspectives. First, we assessed the degree of niche overlap between *A. solaris* and *Acropora* coralis using niche similarity testing. A higher degree of overlap indicates a greater potential impact on coral reefs [71]. Specifically, we measured environmental niche overlap between species using Schoener’s D metric (D), the I similarity statistic (I), and niche equivalency tests [72,73]. Both D and I are classical metrics for evaluating the ecological similarity between populations [74,75,76,77], with values ranging from 0 to 1, where 1 indicates that the two species share the exact same ecological space [72,73]. We also conducted a niche equivalency test to evaluate the identity of the two species in the ecological niche space [78]. This test compared the observed niche overlap of a pair of species with a distribution of 100 random overlap values. The evaluation criterion was the *p*-value, where a significant difference (*p* ≤ 0.05) between observed overlap and random distribution suggested low niche similarity, while a non-significant result indicated potential niche similarity [78]. The analysis was conducted based on the actual distribution of the two species under the current scenario and was performed using the “ecospat” R package version 4.0.0 [79].

Subsequently, we assessed the distribution range overlap between *A. solaris* and *Acropora* corals using the spatial overlap percentage. A higher overlap indicates a greater potential impact on coral reefs. We used the SDMtoolbox toolkit for post-processing species distribution model data [80] and employed its distribution change tool (“the distribution changes between binary SDMs”) to analyze changes in potential habitats/suitable areas over time. Additionally, spatial connectivity and zonal statistical tools were used to calculate the spatial overlap ratio between *A. solaris* and *Acropora* coralis under different climate change scenarios. All analyses were performed using ArcGIS 10.2.

## 3. Results

### 3.1. Current Distribution of A. Solaris and Its Determining Factors

**Figure 2:**
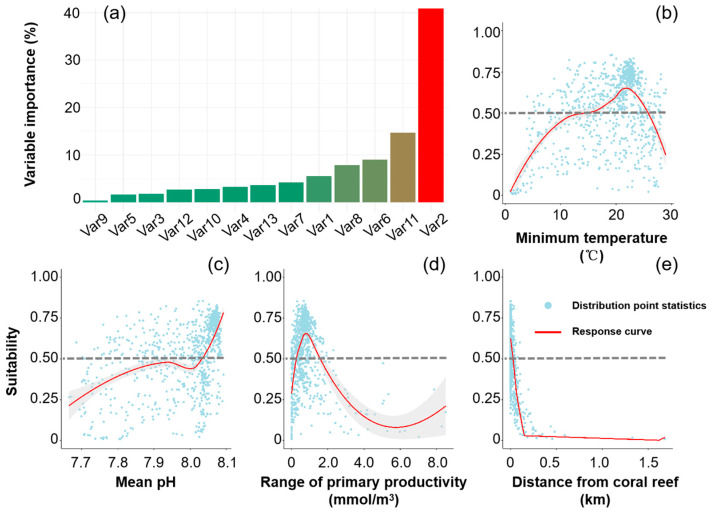
Importance of environmental variables and response curves in the model. (**a**) The permutation importance of environmental variables; (**b**–**e**) the response curves between habitat suitability and the important environmental variables.

The AUC value of the SDMs was 0.907 ± 0.003, and the TSS value was 0.756 ± 0.002, indicating strong model performance and stable results. The permutation importance of the environmental variables revealed that out of the thirteen variables, four were primary factors influencing the distribution of *A. solaris*, accounting for over 70% of the cumulative importance (Figure 2a). The variable with the highest importance was distance from coral reefs (Var2), contributing 42.7%, followed by minimum temperature (Var11), mean pH (Var6), and range of primary productivity (Var8), which accounted for 14.7%, 9.0%, and 7.8%, respectively (Figure 2a). Habitat suitability–environmental variable response curves (Figure 2b–e) showed that habitat suitability for *A. solaris* decreases as the distance from *Acropora* corals increases, with suitable habitats found within 0.1 km of such reefs. Habitat suitability increased with higher pH values, with conditions exceeding a pH of 7.9 deemed suitable. The relationships among the range of primary productivity, minimum temperature, and habitat suitability followed unimodal patterns. The optimal range for primary productivity was between 0.38 and 1.76 mmol/m^3^, while the optimal minimum temperature was between 15 °C and 25 °C.

**Figure 3:**
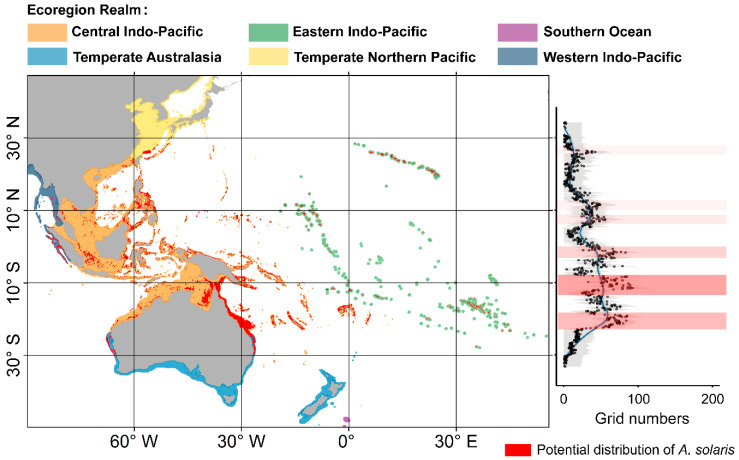
Potential distribution of *A. solaris* under the current scenario, with the red band in the distribution curve on the right representing areas of concentrated distribution.

Under the current scenario, the total distribution area of *A. solaris* was 1,569,807 km^2^, primarily concentrated in the Central Indo-Pacific. The species was highly concentrated in three provinces: the Western Coral Triangle, Northeast Australian Shelf, and Sahul Shelf, which together account for >50% of the total area. In contrast, no suitable habitats for *A. solaris* were identified in five provinces: the Cold Temperate Northwest Pacific, Southeast Australian Shelf, southern New Zealand, Southwest Australian Shelf, and Subantarctic New Zealand (Table 2). Moreover, the distribution of *A. solaris* exhibits strong regional characteristics, with hotspots primarily located near 11° N, 7° N, 2° S, 11° S, and 20° S (Figure 3). These regions include the tropical and subtropical coastal areas of the Philippines, Sulawesi Island in Indonesia, the northeastern coast of Australia (Great Barrier Reef region), and the waters surrounding Papua New Guinea.

### 3.2. Distribution of A. solaris Under Climate Change

Under the influence of climate change, by 2100, the potential suitable habitat for *A. solaris* is projected to decrease by 4.14% under the SSP1-2.6 scenario, reducing the area to 1,504,853 km^2^. In contrast, under SSP2-4.5 and SSP5-8.5, the habitat is expected to increase by 10.28% and 36.50%, reaching 1,731,142 km^2^ and 2,142,710 km^2^, respectively. Regarding latitudinal and longitudinal distributions, *A. solaris* will primarily expand towards higher latitudes, with minimal changes in its longitudinal range. Currently, the suitable habitat spans from 34.32° N to 33.25° S. Under the SSP1-2.6 scenario, the southern boundary shifts southward by 0.08°, whereas under the SSP2-4.5 scenario, both the northern and southern boundaries move poleward by 0.08°. Under the SSP5-8.5, the southern boundary shifts by 4.84° poleward, whereas the northern boundary moves by 0.77° (Table 3). Overall, as climate change progresses, the latitudinal distribution of *A. solaris* is expected to expand asymmetrically, with the northern boundary moving more slowly (approximately 84.7 km) and the southern boundary expanding more rapidly (up to 492.8 km).

**Figure 4:**
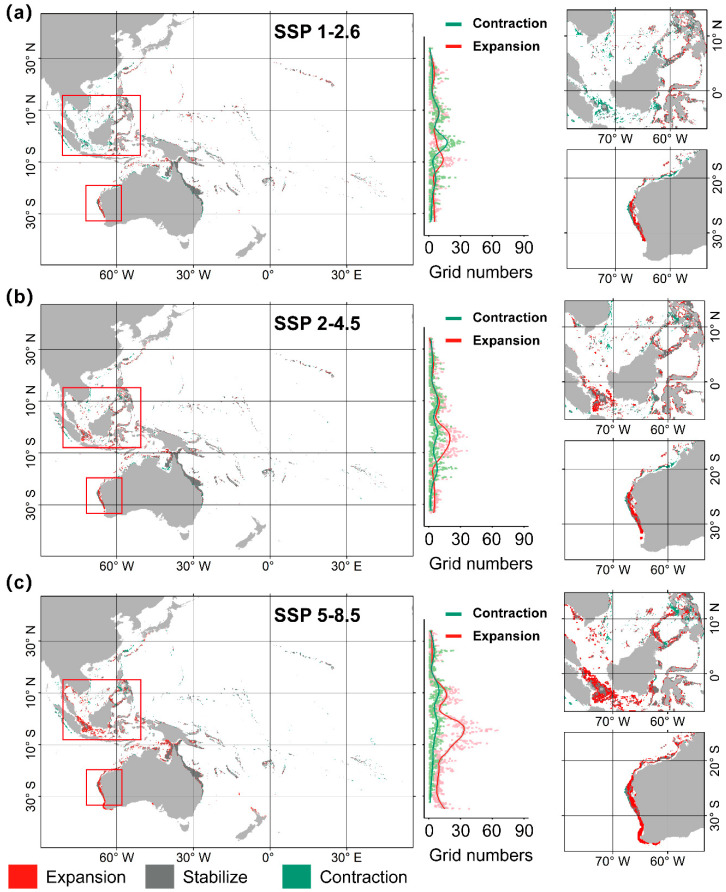
Distribution changes of *A. solaris* under future climate scenarios. (**a**) SSP1-2.6; (**b**) SSP2-4.5; (**c**) SSP5-8.5.

Further analysis of the spatial contraction and expansion trends of *A. solaris* at different latitudes revealed that under the SSP1-2.6 scenario, the most significant habitat contraction occurs near the Equator, while notable habitat expansion is projected around 10° S, in southern Indonesia and northern Australia (Figure 4a). Under the SSP2-4.5 scenario, the greatest contraction is expected around 20°S, with widespread habitat expansion occurring on both sides at 10° S (Figure 4b). The trends under the SSP5-8.5 scenario closely resemble those under the SSP2-4.5 scenario, but with more pronounced shifts. New areas of expansion also emerge north of 30° N and south of 30° S (Figure 4c). In terms of ecoregions (Table 4), the distribution of *A. solaris* will expand to seven provinces, including the Eastern Coral Triangle, South Kuroshio, and West Central Australian Shelf, across all climate scenarios. Conversely, the distribution of species will decrease in five provinces, including the Andaman Sea, Bay of Bengal, and East Central Australian Shelf. Notably, regions such as northern New Zealand, southern New Zealand, and the Southwest Australian Shelf, where *A. solaris* has not been previously recorded, may experience species invasion, whereas its presence in the Bay of Bengal may disappear.

### 3.3. Impact of A. solaris on Acropora Habitats

**Figure 5:**
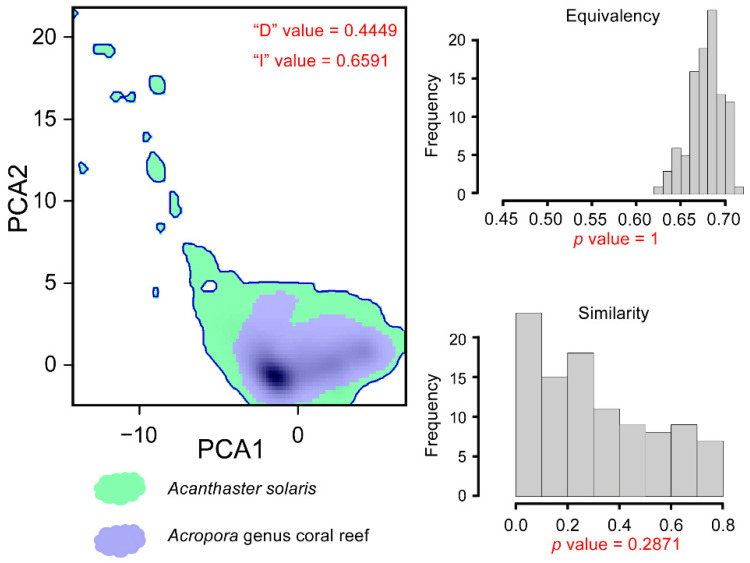
Niche similarity analysis results.

**Figure 6:**
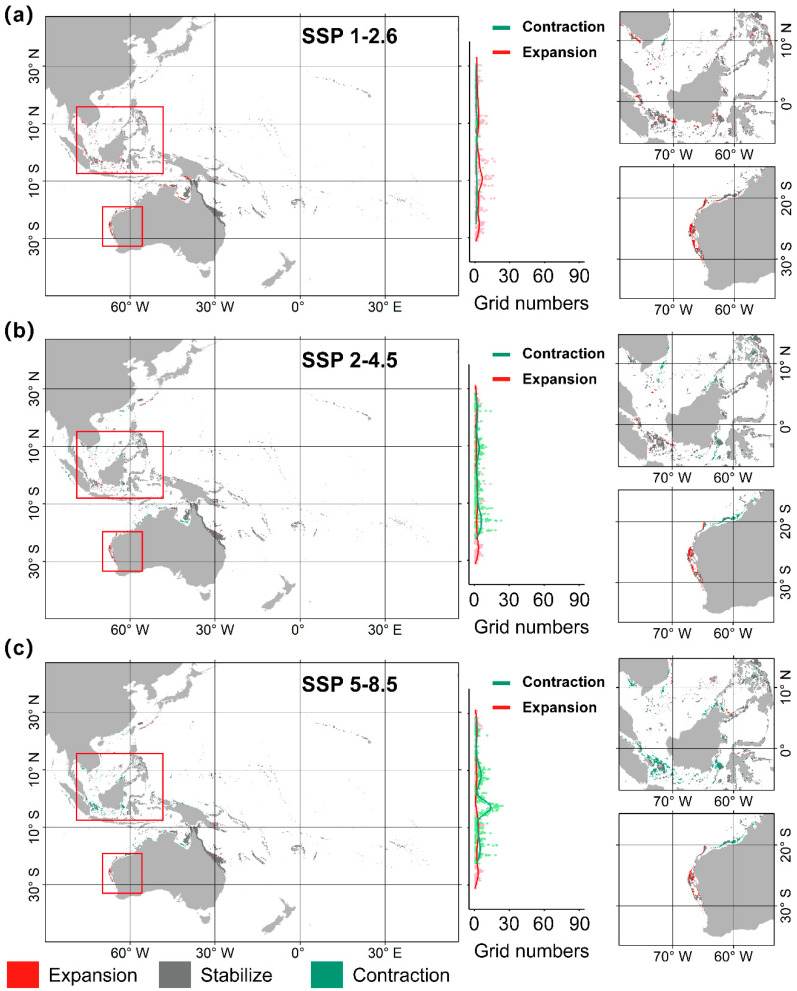
Distribution changes of *Acropora* habitats under future climate scenarios. (**a**) SSP1-2.6; (**b**) SSP2-4.5; (**c**) SSP5-8.5.

Niche overlap calculations showed a D value of 0.45 and an I value of 0.66 between *A. solaris* and *Acropora*, indicating a relatively high environmental niche overlap between the two species (Figure 5). An equivalence test, with *p* < 0.05, further supported the presence of ecological niche similarities. Additionally, the principal component analysis suggests that the environmental niche of *A. solaris* may be slightly broader than that of *Acropora*. These findings suggest that, under climate change, similar environmental niches of the two species could lead to comparable shifts in their distributions. Consequently, the predatory impact of *A. solaris* on *Acropora* may persist in these new areas. In addition to the distribution changes of *A. solaris*, our predictions also indicate a shift in the distribution of *Acropora* habitats (Figure 6), decreasing from the current 1,407,884 km^2^ to between 1,203,780 km^2^ and 1,423,597 km^2^ by 2100. This represents a reduction of -1.1% to 14.5%, with the SSP5-8.5 scenario showing the largest decline.

Further analysis of the distribution of *A. solaris* within *Acropora* habitats showed that, under the current scenario, the overlapping area was 1,135,600 km^2^, representing approximately 80.66% of *Acropora* habitats in the study area (Table 5). Under the SSP1-2.6 scenario for 2100, the overlap area is projected to decrease to 1,032,678 km^2^, or approximately 72.54% of the *Acropora* habitat; under the SSP2-4.5 scenario, it is expected to increase to 1,136,182 km^2^, or approximately 85.96%; and under the SSP5-8.5 scenario, it will further increase to 1,120,119 km^2^, or approximately 93.05%. These results suggest that, under sustainable development scenarios, the impact of *A. solaris* on coral reefs may diminish. However, as climate change intensifies, the impact on species will increase significantly. Among the different ecological regions, the Western Coral Triangle, Northeast Australian Shelf, Sahul Shelf, and Sunda Shelf are expected to face the highest risks, with the Sahul and Sunda Shelves experiencing the most rapid increases in risk.

## 4. Discussion

### 4.1. Distribution and Dynamics of A. solaris

We found that the main factors influencing the distribution of *A. solaris* include the distance from coral reefs, sea temperature, pH, and primary productivity. As an obligate coral-associated species, the distribution pattern of *A. solaris* is strongly correlated with coral reefs. *A. solaris* feeds on corals, particularly branching corals of the *Acropora* genus. Coral tissues serve as a crucial nutritional source for both survival and reproduction, and coral reefs provide *A. solaris* with refuge from predators and breeding grounds [28,29,48]. Environmental factors, such as sea temperature, pH, and primary productivity, have been shown to be closely linked to the reproduction, growth, and catastrophic outbreaks of *A. solaris* larvae [38,81,82,83]. Studies have indicated that excessively high or low environmental temperatures can affect the reproduction and development of *A. solaris* and even affect the survival rate of individuals after maturation [82,83,84,85]. Ocean acidification (pH < 7.75) has been shown to cause delays or even hinder the development of benthic larvae [82]. Additionally, a study on the survival and settlement of *A. solaris* larvae at varying food concentrations found that both excessively high and low food concentrations were detrimental to larval survival and settlement [81].

In this study, we present the first potentially suitable habitat distribution map for *A. solaris*. Hotspots for this species are concentrated near 11° N, 7° N, 2° S, 11° S, and 20° S. According to the available records, outbreaks of *A. solaris* have been reported in these regions, with the majority of reports concentrated on the Great Barrier Reef, New Guinea, Indonesia, and Malaysia [12,16,26,27,86,87,88]. Outbreaks have also been recorded in Japan, Guam, and the Ryukyu Islands [4]. Under the influence of climate change, we predict that by 2100, the northern range of *A. solaris* will expand by a maximum equivalent distance of approximately 84.7 km, while the southern range will expand by a maximum of approximately 492.8 km, with an average poleward movement distance of 288.4 km. Previous studies have predicted an average poleward shift of approximately 256 km in global marine invertebrate distributions under climate change (RCP8.5 scenario), which is consistent with our prediction results [89].

### 4.2. Risk of A. solaris Under Climate Change

This study found that *A. solaris* exhibits a high environmental niche overlap with *Acropora*, but that the environmental niche of *A. solaris* may be slightly broader than that of *Acropora*. This suggests that *A. solaris* may have a stronger adaptive capacity to various marine environments, allowing it to thrive under a wider range of environmental conditions than can *Acropora*. Previous studies have shown that the settlement rate of *A. solaris* larvae is highly resistant to ocean warming [85], with high settlement success and survival rates at temperatures below 32 °C. In contrast, *Acropora* larvae are highly sensitive to temperature, with a significant decrease in the survival rate above 30 °C [90,91]. This suggests that, as climate change drives shifts in marine environments (such as rising water temperatures and increased acidification), the growth and expansion potential of *A. solaris* could be further enhanced, particularly in tropical and subtropical regions. In terms of its feeding niche, *A. solaris* primarily consumes coral tissue, with a strong preference for *Acropora* species. However, in non-reef habitats, where coral prey is scarce, or under experimental conditions, *A. solaris* may occasionally feed on other available marine organisms, such as soft corals, sponges, mollusks, and even conspecifics [15,16,48]. This suggests that, under specific conditions, *A. solaris* can survive in non-reef environments, such as lagoon habitats or areas with scattered coral patches [24]. Nevertheless, these alternative food sources are insufficient to sustain large populations, meaning that their survival and population growth remain heavily dependent on the availability of coral. As a result, *A. solaris* struggles to reproduce in areas completely devoid of coral reefs. With rising temperatures, the population density and reproductive frequency of *A. solaris* may increase, and higher densities will likely continue to concentrate in coral reef habitats, leading to an increased potential for outbreaks and subsequent ecological damage [92,93].

Additionally, we found that climate change could facilitate the migration of *A. solaris* into previously unaffected areas, thereby increasing the risk of biological invasion into these new ecosystems and threatening local coral reef ecosystems. This risk is particularly pronounced when these new areas lack natural predators because the spread of *A. solaris* may trigger a cascade of chain reactions along the food web, ultimately leading to the collapse of coral reef ecosystems [94]. Many coral reefs face risks from both climate change and predation [95,96,97]. Climate change may alter coral growth patterns, reproductive capabilities, and stress resistance, whereas the invasion of predators like *A. solaris* exacerbates the survival pressure on corals. When corals are under physiological stress due to rising temperatures or other environmental changes, they may become more vulnerable to predation [98], accelerating coral reef degradation. For example, a decade-long observation of coral recovery after the outbreak of *A.* cf. *solaris* in the Great Barrier Reef revealed that while most reefs recovered with positive growth rates, recurrent climate events continued to limit their full recovery [99]. Therefore, the dual risks of climate change and predation pressure further exacerbate the degradation of coral reefs, affecting related industries such as fisheries, tourism, and coastal protection [100]. This highlights the need for urgent and multidimensional conservation measures that address the environmental pressures induced by climate change and control the spread of harmful species [83,101,102].

### 4.3. Study Uncertainty and Limitations

Despite the effective prediction of the long-term distribution changes of *A. solaris* in our study, certain limitations remain. The selection of environmental variables was based on their known ecological relevance to *A. solaris*, but there may be other unmeasured factors (e.g., biotic interactions) that could influence its distribution. For instance, machine learning models do not account for potential changes in coral reef health, which could alter the distribution patterns of *A. solaris* [11,103]. The effect of *A. solaris* on coral reefs may be influenced by the presence of natural predators (e.g., Lethrinidae and Balistidae fishes) or competitors that could mitigate or exacerbate its effects [104,105]. Furthermore, this study focuses on 2100, which is a long-term projection. The distribution of *A. solaris* may change at different rates over shorter timescales; however, our predictions did not account for potential intermediate changes or tipping points in the distribution of the species.

Our study also contains certain uncertainties, primarily stemming from potential changes in the adaptive capacity and population dynamics of *A. solaris*. The prediction model assumed that *A. solaris* will respond passively to environmental changes. However, species may exhibit adaptive behaviors or physiological changes in response to shifting environmental conditions, which could alter their distribution patterns [106,107]. Additionally, the potential for genetic adaptation or phenotypic plasticity was not considered in this study [108]. Furthermore, we assumed a static relationship between *A. solaris* and its environment, and in assessing its impact, we only considered the environmental ecological niches and spatial distributions of *A. solaris* and *Acropora*. However, in reality, the distribution of *A. solaris* and its outbreak events may be influenced by dynamic processes such as larval dispersal, population dynamics, and seasonal variations [6,81,85]. These factors can affect the accuracy of long-term impact projections.

## 5. Conclusions

This study integrated both non-biological and biological predictors to project the changes in the distribution of *A. solaris* under climate change. We incorporated the distribution of *Acropora* corals into the SDM framework as a biological factor by utilizing a two-step approach to predict species distribution. This methodological innovation distinguishes our study from traditional studies that rely solely on abiotic factors. Our study also provides the first reliable set of current and future distribution maps for *A. solaris* created using a comprehensive global dataset and machine learning model. The results indicate that under climate change, *A. solaris* will experience a range of shifts, with notable expansion in certain regions and contraction in others, particularly near the Equator. The overlap between *A. solaris* and *Acropora* habitats is particularly concerning, as it suggests that the species may increase its predatory impact in areas such as the Western Coral Triangle and Northeast Australian Shelf. Furthermore, regions previously uninhabited by *A. solaris*, such as parts of New Zealand, may face new invasions. This study provides critical data for understanding the ecological dynamics of this species in the context of climate change. Moreover, this study has important implications for coral reef conservation and management, especially in regions predicted to face rapid ecological shifts. Future research should focus on developing mitigation strategies to protect coral reef ecosystems from the growing threat posed by *A. solaris*.

## Figures and Tables

**Table 1:** Abiotic and biotic variables and sources.

Variable	Type	Acronym	Unit	ID	Source
Bathymetry	Abiotic	Bathymetry	meter	Var1	gebco.net
Distance from coral reef	Biotic	Dis	meter	Var2	SDM outputs
Mean dissolved oxygen	Abiotic	DO_mean	mmol/m^3^	Var3	Bio-ORACLE v3.0 (https://www.bio-oracle.org/, accessed on 15 July 2024 [50])
Mean diffuse attenuation	Abiotic	Kdpar_mean	m^−1^	Var4	
Range of nitrate	Abiotic	N_range	mmol/m^3^	Var5	
Mean pH	Abiotic	PH_mean	-	Var6	
Minimum primary productivity	Abiotic	PP_mean	mmol/m^3^	Var7	
Range primary productivity	Abiotic	PP_range	mmol/m^3^	Var8	
Maximum salinity	Abiotic	S_max	mmol/m^3^	Var9	
Range of salinity	Abiotic	S_range	mmol/m^3^	Var10	
Minimum temperature	Abiotic	T_min	°C	Var11	
Range of temperature	Abiotic	T_range	°C	Var12	
Mean current velocity	Abiotic	Velocity_mean	m/s	Var13	

**Table 2:** Habitat area within different ecoregions (km^2^).

Realm	Province	Area (km^2^)	Percentage
Western Indo-Pacific	Andaman	46,887	2.99%
Western Indo-Pacific	Bay of Bengal	252	0.02%
Eastern Indo-Pacific	Central Polynesia	7310	0.47%
Temperate Northern Pacific	Cold Temperate Northwest Pacific	0	0.00%
Temperate Australasia	East Central Australian Shelf	41,173	2.62%
Central Indo-Pacific	Eastern Coral Triangle	121,252	7.72%
Eastern Indo-Pacific	Hawaii	27,729	1.77%
Central Indo-Pacific	Java Transitional	3361	0.21%
Central Indo-Pacific	Lord Howe and Norfolk Islands	1764	0.11%
Eastern Indo-Pacific	Marquesas	2016	0.13%
Eastern Indo-Pacific	Marshall, Gilbert, and Ellis Islands	35,123	2.24%
Central Indo-Pacific	Northeast Australian Shelf	264,855	16.87%
Temperate Australasia	Northern New Zealand	84	0.01%
Central Indo-Pacific	Northwest Australian Shelf	37,980	2.42%
Central Indo-Pacific	Sahul Shelf	182,424	11.62%
Central Indo-Pacific	South China Sea	41,089	2.62%
Central Indo-Pacific	South Kuroshio	19,914	1.27%
Temperate Australasia	Southeast Australian Shelf	0	0.00%
Eastern Indo-Pacific	Southeast Polynesia	31,930	2.03%
Temperate Australasia	Southern New Zealand	0	0.00%
Temperate Australasia	Southwest Australian Shelf	0	0.00%
Southern Ocean	Subantarctic New Zealand	0	0.00%
Central Indo-Pacific	Sunda Shelf	122,932	7.83%
Central Indo-Pacific	Tropical Northwestern Pacific	39,913	2.54%
Central Indo-Pacific	Tropical Southwestern Pacific	134,192	8.55%
Temperate Northern Pacific	Warm Temperate Northwest Pacific	31,594	2.01%
Temperate Australasia	West Central Australian Shelf	35,711	2.27%
Central Indo-Pacific	Western Coral Triangle	340,312	21.68%
Total	\	1,569,806	100.00%

**Table 3:** Latitudinal distribution range shifts of *A. solaris*.

Scenario	North Latitude	South Latitude	Northward Shift	Southward Shift
Present	34.31°	33.26°	\	\
SSP1-2.6	34.31°	33.34°	0°	0.08°
SSP2-4.5	34.41°	33.34°	0.08°	0.08°
SSP5-8.5	35.08°	38.09°	0.77°	4.84°

**Table 4:** Habitat area of *A. solaris* in different ecological zones under future climate scenarios (km^2^).

REALM	PROVINC	SSP1-2.6	SSP2-4.5	SSP5-8.5
Western Indo-Pacific	Andaman	10,503	17,394	14,033
Western Indo-Pacific	Bay of Bengal	84	0	0
Eastern Indo-Pacific	Central Polynesia	7478	6386	4790
Temperate Northern Pacific	Cold Temperate Northwest Pacific	0	0	0
Temperate Australasia	East Central Australian Shelf	30,670	37,308	40,081
Central Indo-Pacific	Eastern Coral Triangle	121,336	126,378	127,722
Eastern Indo-Pacific	Hawaii	28,990	27,897	27,057
Central Indo-Pacific	Java Transitional	2605	7731	4790
Central Indo-Pacific	Lord Howe and Norfolk Islands	2353	2269	4537
Eastern Indo-Pacific	Marquesas	2101	1512	588
Eastern Indo-Pacific	Marshall, Gilbert and Ellis Islands	32,519	34,872	30,166
Central Indo-Pacific	Northeast Australian Shelf	256,621	264,772	280,989
Temperate Australasia	Northern New Zealand	0	0	8991
Central Indo-Pacific	Northwest Australian Shelf	35,292	37,897	80,835
Central Indo-Pacific	Sahul Shelf	196,541	195,197	300,063
Central Indo-Pacific	South China Sea	28,149	40,417	50,249
Central Indo-Pacific	South Kuroshio	24,536	25,376	25,208
Temperate Australasia	Southeast Australian Shelf	0	0	0
Eastern Indo-Pacific	Southeast Polynesia	33,359	30,670	28,317
Temperate Australasia	Southern New Zealand	0	0	3949
Temperate Australasia	Southwest Australian Shelf	0	2017	39,157
Southern Ocean	Subantarctic New Zealand	0	0	0
Central Indo-Pacific	Sunda Shelf	52,769	174,106	340,901
Central Indo-Pacific	Tropical Northwestern Pacific	43,526	38,569	33,779
Central Indo-Pacific	Tropical Southwestern Pacific	140,578	136,377	139,486
Temperate Northern Pacific	Warm Temperate Northwest Pacific	26,049	31,258	26,973
Temperate Australasia	West Central Australian Shelf	66,046	74,701	86,633
Central Indo-Pacific	Western Coral Triangle	362,748	418,038	443,415

**Table 5:** Overlap of *A. solaris* and *Acropora* coral habitats under different climate change scenarios (km^2^).

Scenario	Habitat Area (*A. solaris*)	Habitat Area (*Acropora*)	Overlap	Occupancy Rate of *A. solaris* in *Acropora* Habitats
Present	1,569,807	1,407,884	1,135,600	80.66%
SSP1-2.6	1,504,853	1,423,597	1,032,678	72.54%
SSP2-4.5	1,731,142	1,321,755	1,136,182	85.96%
SSP5-8.5	2,142,710	1,203,780	1,120,119	93.05%

## Data Availability

The original species distribution data were derived from the following resources available in the public domain: the Global Biodiversity Information Facility (https://www.gbif.org/, accessed on 14 July 2024), Ocean Biodiversity Information System (https://obis.org/, accessed on 14 July 2024), iNaturalist (https://www.inaturalist.org/, accessed on 14 July 2024), and Atlas of Living Australia (https://www.ala.org.au/, accessed on 14 July 2024). Filtered species distribution data supporting the findings of this study are available from the corresponding author on request. The environmental data for habitat suitability modeling are available at: https://www.bio-oracle.org/downloads-to-email.php (accessed on 14 July 2024), and gebco.net.

## Supplementary Materials

The following supporting information can be downloaded at: https://www.mdpi.com/article/10.3390/ani15060858/s1, Figure S1: Results of the correlation analysis of variables; Table S1: Alternative abiotic factors; Table S2: ODMAP (Overview, Data, Model, Assessment, Prediction) protocol.

## References

[1] Fabricius K.E., Okaji K., De’ath G. (2010). Three lines of evidence to link outbreaks of the crown-of-thorns seastar *Acanthaster planci* to the release of larval food limitation. Coral Reefs.

[2] Wang Y., Gu Y.-B., Guo H., Cao L.-Q., Jin Y. (2023). Advances and perspectives on the research of starfish outbreaks in northern China. Ying Yong Sheng Tai Xue Bao.

[3] Vogler C., Benzie J., Lessios H., Barber P., Wörheide G. (2008). A threat to coral reefs multiplied? Four species of crown-of-thorns starfish. Biol. Lett..

[4] Chesher R.H. (1969). Destruction of Pacific Corals by the Sea Star *Acanthaster planci*. Science.

[5] Adams M.S., Demmig-Adams B., Li R., Zarate D., Li J. (2020). Coral reef productivity and diversity—Contributions from enhanced photosynthesis via demand for carbohydrate from the host. Mar. Ecol..

[6] Pratchett M.S., Caballes C.F., Wilmes J.C., Matthews S., Mellin C., Sweatman H.P.A., Nadler L.E., Brodie J., Thompson C.A., Hoey J. (2017). Thirty Years of Research on Crown-of-Thorns Starfish (1986–2016): Scientific Advances and Emerging Opportunities. Diversity.

[7] Carpenter K.E., Abrar M., Aeby G., Aronson R.B., Banks S., Bruckner A., Chiriboga A., Cortés J., Delbeek J.C., DeVantier L. (2008). One-Third of Reef-Building Corals Face Elevated Extinction Risk from Climate Change and Local Impacts. Science.

[8] Uthicke S., Pratchett M.S., Bronstein O., Alvarado J.J., Wörheide G. (2024). The crown-of-thorns seastar species complex: Knowledge on the biology and ecology of five corallivorous *Acanthaster* species. Mar. Biol..

[9] De’ath G., Fabricius K.E., Sweatman H., Puotinen M. (2012). The 27–year decline of coral cover on the Great Barrier Reef and its causes. Proc. Natl. Acad. Sci. USA.

[10] Castro-Sanguino C., Bozec Y.-M., Condie S.A., Fletcher C.S., Hock K., Roelfsema C., Westcott D.A., Mumby P.J. (2023). Control efforts of crown-of-thorns starfish outbreaks to limit future coral decline across the Great Barrier Reef. Ecosphere.

[11] Keesing J.K., Thomson D.P., Haywood M.D.E., Babcock R.C. (2019). Two time losers: Selective feeding by crown-of-thorns starfish on corals most affected by successive coral-bleaching episodes on western Australian coral reefs. Mar. Biol..

[12] Baird A.H., Pratchett M.S., Hoey A.S., Herdiana Y., Campbell S.J. (2013). *Acanthaster planci* is a major cause of coral mortality in Indonesia. Coral Reefs.

[13] Foo S.A., Millican H.R., Byrne M. (2024). Crown-of-thorns seastar (*Acanthaster* spp.) feeding ecology across species and regions. Sci. Total Environ..

[14] Neil R.C., Gomez Cabrera M., Uthicke S. (2022). Juvenile age and available coral species modulate transition probability from herbivory to corallivory in *Acanthaster* cf. Solaris (Crown-of-Thorns Seastar). Coral Reefs.

[15] Chansang H., Boonyanate P., Pongsuwan N., Charuchinda M., Wungboonkong G. (1987). Infestation of *Acanthaster*-Planci in the Andaman Sea. Bull. Mar. Sci..

[16] De’ath G., Moran P.J. (1998). Factors affecting the behaviour of crown-of-thorns starfish (*Acanthaster planci* L.) on the Great Barrier Reef: 2: Feeding preferences. J. Exp. Mar. Biol. Ecol..

[17] Hunt J. (2013). Great Barrier Reef coral loss and crown-of-thorns starfish. Aust. Canegrower.

[18] Munday P.L. (2004). Habitat loss, resource specialization, and extinction on coral reefs. Glob. Change Biol..

[19] Osborne K., Dolman A.M., Burgess S.C., Johns K.A. (2011). Disturbance and the Dynamics of Coral Cover on the Great Barrier Reef (1995–2009). PLoS ONE.

[20] Pratchett M.S., Caballes C.F., Rivera-Posada J.A., Sweatman H.P.A. (2014). Limits to understanding and managing outbreaks of crown-of-thorns starfish (*Acanthaster* spp.). Oceanogr. Mar. Biol. Annu. Rev..

[21] Uthicke S., Robson B., Doyle J.R., Logan M., Pratchett M.S., Lamare M. (2022). Developing an effective marine eDNA monitoring: eDNA detection at pre-outbreak densities of corallivorous seastar (*Acanthaster* cf. Solaris). Sci. Total Environ..

[22] Peng C., Wang K., Wang W., Kuang F., Gao Y., Jiang R., Sun X., Dong X., Chen B., Lin H. (2023). Phytoplankton community structure and environmental factors during the outbreak of Crown-of-Thorns Starfish in Xisha Islands, South China Sea. Environ. Res..

[23] Yan Z., Xing J., Cai W., Zhang K., Wu Z., Li Y., Tang J., Zhou Z. (2023). Study on the population distribution of Acanthaster planci in the reef area of the Xisha Islands based on environmental DNA technology. Haiyang Xuebao.

[24] Tkachenko K.S., Hoang D.T. (2022). Concurrent effect of crown-of-thorns starfish outbreak and thermal anomaly of 2020 on coral reef communities of the Spratly Islands (South China Sea). Mar. Ecol..

[25] Trapon M.L., Pratchett M.S., Penin L. (2011). Comparative Effects of Different Disturbances in Coral Reef Habitats in Moorea, French Polynesia. J. Mar. Sci..

[26] Plass-Johnson J.G., Schwieder H., Heiden J., Weiand L., Wild C., Jompa J., Ferse S.C.A., Teichberg M. (2015). A recent outbreak of crown-of-thorns starfish (*Acanthaster planci*) in the Spermonde Archipelago, Indonesia. Reg. Environ. Change.

[27] Chak S.T.C., Dumont C.P., Adzis K.-A.A., Yewdall K. (2018). Effectiveness of the removal of coral-eating predator *Acanthaster planci* in Pulau Tioman Marine Park, Malaysia. J. Mar. Biol. Assoc. UK.

[28] Birkeland C., Lucas J. (1990). Acanthaster planci: Major Management Problem of Coral Reefs.

[29] Pratchett M.S., Lang B.J., Matthews S. (2019). Culling crown-of-thorns starfish (*Acanthaster* cf. Solaris) on Australia’s Great Barrier Reef: Rationale and effectiveness. Aust. Zool..

[30] Kamya P.Z., Dworjanyn S.A., Hardy N., Mos B., Uthicke S., Byrne M. (2014). Larvae of the coral eating crown-of-thorns starfish, Acanthaster planci, in a warmer–high CO_2_ ocean. Glob. Change Biol..

[31] Kamya P.Z., Byrne M., Graba-Landry A., Dworjanyn S.A. (2016). Near-future ocean acidification enhances the feeding rate and development of the herbivorous juveniles of the crown-of-thorns starfish, Acanthaster planci. Coral Reefs..

[32] Yasuda N., Iguchi A., Hongo C. (2018). Distribution Expansion and Historical Population Outbreak Patterns of Crown-of-Thorns Starfish, Acanthaster planci Sensu Lato, in Japan from 1912 to 2015.

[33] Lane D.J.W. (2012). *Acanthaster planci* impact on coral communities at permanent transect sites on Bruneian reefs, with a regional overview and a critique on outbreak causes. J. Mar. Biol. Assoc. UK.

[34] Tkachenko K.S., Huan N.H., Thanh N.H., Britayev T.A. (2021). Extensive coral reef decline in Nha Trang Bay, Vietnam: *Acanthaster planci* outbreak: The final event in a sequence of chronic disturbances. Mar. Freshw. Res..

[35] Tkachenko K.S. (2023). Degradation of Coral Reefs under Complex Impact of Natural and Anthropogenic Factors with Nha Trang Bay (Vietnam) as an Example. Biol. Bull. Rev..

[36] Glynn P.W. (1968). Mass mortalities of echinoids and other reef flat organisms coincident with midday, low water exposures in Puerto Rico. Mar. Biol..

[37] Dahlke F.T., Wohlrab S., Butzin M., Pörtner H.-O. (2020). Thermal bottlenecks in the life cycle define climate vulnerability of fish. Science.

[38] Sill S.R., Dawson T.P. (2021). Climate change impacts on the ecological dynamics of two coral reef species, the humphead wrasse (*Cheilinus undulatus*) and crown-of-thorns starfish (*Ancanthaster planci*). Ecol. Inform..

[39] Hue T., Chateau O., Lecellier G., Marin C., Coulombier N., Le Dean L., Gossuin H., Adjeroud M., Dumas P. (2022). Impact of near-future ocean warming and acidification on the larval development of coral-eating starfish *Acanthaster* Solaris after parental exposure. J. Exp. Mar. Biol. Ecol..

[40] Simon T.N., Levitan D.R. (2011). Measuring Fertilization Success of Broadcast-Spawning Marine Invertebrates Within Seagrass Meadows. Biol. Bull..

[41] Yamano H., Sugihara K., Nomura K. (2011). Rapid poleward range expansion of tropical reef corals in response to rising sea surface temperatures: Poleward Range Expansion of Corals. Geophys. Res. Lett..

[42] Xu Y., Ma L., Sui J., Li X., Wang H., Zhang B. (2023). Potential impacts of climate change on the distribution of echinoderms in the Yellow Sea and East China Sea. Mar. Pollut. Bull..

[43] Koop K., Booth D., Dennison W., Erdmann M., Jones G.B., Larkum A.W.D., O’Neil J., Steven A., Tentori E., Ward S. (2001). Encore: The Effect of Nutrient Enrichment on Coral Reefs. Synthesis of Results and Conclusions. Mar. Pollut. Bull..

[44] Anthony K.R.N., Kline D.I., Diaz-Pulido G., Dove S., Hoegh-Guldberg O. (2008). Ocean acidification causes bleaching and productivity loss in coral reef builders. Proc. Natl. Acad. Sci. USA.

[45] Despalatović M., Grubelić I., Piccinetti C., Cvitković I., Antolić B., Žuljević A., Nikolić V. (2009). Distribution of echinoderms on continental shelf in open waters of the northern and middle Adriatic Sea. J. Mar. Biol. Assoc. UK.

[46] Stuart-Smith R.D., Bates A.E., Lefcheck J.S., Duffy J.E., Baker S.C., Thomson R.J., Stuart-Smith J.F., Hill N.A., Kininmonth S.J., Airoldi L. (2013). Integrating abundance and functional traits reveals new global hotspots of fish diversity. Nature.

[47] Pyle R.L., Copus J.M., Loya Y., Puglise K., Bridge T. (2019). Mesophotic Coral Ecosystems: Introduction and Overview.

[48] Barnes M. (1986). The *Acanthaster* Phenomenon. Oceanogr. Mar. Biol..

[49] Lucas J.S. (2013). Crown-of-thorns starfish. Curr. Biol..

[50] Assis J., Fernández Bejarano S.J., Salazar V.W., Schepers L., Gouvêa L., Fragkopoulou E., Leclercq F., Vanhoorne B., Tyberghein L., Serrão E.A. (2024). Bio-ORACLE v3.0. Pushing marine data layers to the CMIP6 Earth System Models of climate change research. Glob. Ecol. Biogeogr..

[51] (2024). The GEBCO 2024 Grid—A Continuous Terrain Model of the Global Oceans and Land.

[52] Tyberghein L., Verbruggen H., Pauly K., Troupin C., Mineur F., De Clerck O. (2012). Bio-ORACLE: A global environmental dataset for marine species distribution modelling. Glob. Ecol. Biogeogr..

[53] O’Neill B.C., Tebaldi C., Van Vuuren D.P., Eyring V., Friedlingstein P., Hurtt G., Knutti R., Kriegler E., Lamarque J.-F., Lowe J. (2016). The Scenario Model Intercomparison Project (ScenarioMIP) for CMIP6. Geosci. Model Dev..

[54] Dormann C.F., Elith J., Bacher S., Buchmann C., Carl G., Carré G., Marquéz J.R.G., Gruber B., Lafourcade B., Leitão P.J. (2013). Collinearity: A review of methods to deal with it and a simulation study evaluating their performance. Ecography.

[55] Owens H., Barve V., Chamberlain S. (2024). spocc: Interface to Species Occurrence Data Sources. R Package Version 1.2.3. https://docs.ropensci.org/spocc/.

[56] Mammola S., Goodacre S.L., Isaia M. (2018). Climate change may drive cave spiders to extinction. Ecography.

[57] Hu W., Su S., Mohamed H.F., Xiao J., Kang J., Krock B., Xie B., Luo Z., Chen B. (2024). Assessing the global distribution and risk of harmful microalgae: A focus on three toxic Alexandrium dinoflagellates. Sci. Total Environ..

[58] Spalding M.D., Fox H.E., Allen G.R., Davidson N., Ferdaña Z.A., Finlayson M., Halpern B.S., Jorge M.A., Lombana A., Lourie S.A. (2007). Marine Ecoregions of the World: A Bioregionalization of Coastal and Shelf Areas. BioScience.

[59] Zhang Z., Ma S., Bede-Fazekas Á., Mammola S., Qu M., Zhou J., Lin Q. (2024). Considering biotic interactions exacerbates the predicted impacts of climate change on coral-dwelling species. J. Biogeogr..

[60] Elith J., Phillips S.J., Hastie T., Dudík M., Chee Y.E., Yates C.J. (2011). A statistical explanation of MaxEnt for ecologists: Statistical explanation of MaxEnt. Divers. Distrib..

[61] Phillips S.J., Anderson R.P., Dudík M., Schapire R.E., Blair M.E. (2017). Opening the black box: An open-source release of Maxent. Ecography.

[62] Melo-Merino S.M., Reyes-Bonilla H., Lira-Noriega A. (2020). Ecological niche models and species distribution models in marine environments: A literature review and spatial analysis of evidence. Ecol. Model..

[63] Barve N., Barve V., Jiménez-Valverde A., Lira-Noriega A., Maher S.P., Peterson A.T., Soberón J., Villalobos F. (2011). The crucial role of the accessible area in ecological niche modeling and species distribution modeling. Ecol. Model..

[64] Thuiller W., Guéguen M., Renaud J., Karger D.N., Zimmermann N.E. (2019). Uncertainty in ensembles of global biodiversity scenarios. Nat. Commun..

[65] Shipley B.R., Bach R., Do Y., Strathearn H., McGuire J.L., Dilkina B. (2022). megaSDM: Integrating dispersal and time-step analyses into species distribution models. Ecography.

[66] De Kort H., Baguette M., Lenoir J., Stevens V.M. (2020). Toward reliable habitat suitability and accessibility models in an era of multiple environmental stressors. Ecol. Evol..

[67] Swets J.A. (1988). Measuring the Accuracy of Diagnostic Systems. Science.

[68] Allouche O., Tsoar A., Kadmon R. (2006). Assessing the accuracy of species distribution models: Prevalence, kappa and the true skill statistic (TSS). J. Appl. Ecol..

[69] Kass J.M., Muscarella R., Galante P.J., Bohl C.L., Pinilla-Buitrago G.E., Boria R.A., Soley-Guardia M., Anderson R.P. (2021). ENMeval 2.0: Redesigned for customizable and reproducible modeling of species’ niches and distributions. Methods Ecol. Evol..

[70] Zurell D., Franklin J., König C., Bouchet P.J., Dormann C.F., Elith J., Fandos G., Feng X., Guillera-Arroita G., Guisan A. (2020). A standard protocol for reporting species distribution models. Ecography.

[71] Broennimann O., Fitzpatrick M.C., Pearman P.B., Petitpierre B., Pellissier L., Yoccoz N.G., Guisan A. (2012). Measuring ecological niche overlap from occurrence and spatial environmental data. Glob. Ecol. Biogeogr..

[72] Schoener T.W. (1970). Nonsynchronous spatial overlap of lizards in patchy habitats. Ecology.

[73] Warren D.L., Glor R.E., Turelli M. (2008). Environmental Niche Equivalency Versus Conservatism: Quantitative Approaches To Niche Evolution. Evolution.

[74] Encarnación-Luévano A., Escoto-Moreno J.A., Villalobos-Jiménez G. (2022). Evaluating Potential Distribution and Niche Divergence among Populations of the World’s Largest Living Damselfly, *Megaloprepus caerulatus* (Drury, 1782). Diversity.

[75] Smyčka J., Roquet C., Boleda M., Alberti A., Boyer F., Douzet R., Perrier C., Rome M., Valay J.-G., Denoeud F. (2022). Tempo and drivers of plant diversification in the European mountain system. Nat. Commun..

[76] Tong R., Davies A.J., Yesson C., Yu J., Luo Y., Zhang L., Burgos J.M. (2023). Environmental drivers and the distribution of cold-water corals in the global ocean. Front. Mar. Sci..

[77] Zhou Y., Lu X., Zhang G. (2023). Potentially differential impacts on niche overlap between Chinese endangered Zelkova schneideriana and its associated tree species under climate change. Front. Ecol. Evol..

[78] Aguirre-Gutiérrez J., Serna-Chavez H.M., Villalobos-Arambula A.R., Pérez de la Rosa J.A., Raes N. (2015). Similar but not equivalent: Ecological niche comparison across closely—Related M exican white pines. Divers. Distrib..

[79] Broennimann O., Di Cola V., Guisan A., Ecospat: Spatial Ecology Miscellaneous Methods (2023). R Package Version 4.0.0. https://CRAN.R-project.org/package=ecospat.

[80] Brown J.L. (2014). SDM toolbox: A python-based GIS toolkit for landscape genetic, biogeographic and species distribution model analyses. Methods Ecol. Evol..

[81] Pratchett M.S., Dworjanyn S., Mos B., Caballes C.F., Thompson C.A., Blowes S. (2017). Larval Survivorship and Settlement of Crown-of-Thorns Starfish (*Acanthaster* cf. Solaris) at Varying Algal Cell Densities. Diversity.

[82] Hue T., Chateau O., Lecellier G., Kayal M., Lanos N., Gossuin H., Adjeroud M., Dumas P. (2020). Temperature affects the reproductive outputs of coral-eating starfish *Acanthaster* spp. After adult exposure to near-future ocean warming and acidification. Mar. Environ. Res..

[83] Lang B.J., Donelson J.M., Caballes C.F., Uthicke S., Doll P.C., Pratchett M.S. (2022). Effects of elevated temperature on the performance and survival of pacific crown-of-thorns starfish (*Acanthaster* cf. Solaris). Mar. Biol..

[84] Espinel-Velasco N., Hoffmann L., Agüera A., Byrne M., Dupont S., Uthicke S., Webster N.S., Lamare M. (2018). Effects of ocean acidification on the settlement and metamorphosis of marine invertebrate and fish larvae: A review. Mar. Ecol. Prog. Ser..

[85] Lang B.J., Caballes C.F., Uthicke S., Doll P.C., Donelson J.M., Pratchett M.S. (2023). Impacts of ocean warming on the settlement success and post-settlement survival of Pacific crown-of-thorns starfish (*Acanthaster* cf. Solaris). Coral Reefs.

[86] Baine M.S.P. (2006). A major outbreak of crown-of-thorns starfish in Bootless Bay, Central Province, Papua New Guinea. Coral Reefs.

[87] Pratchett M.S., Schenk T.J., Baine M., Syms C., Baird A.H. (2009). Selective coral mortality associated with outbreaks of *Acanthaster planci* L. in Bootless Bay 2009, Papua New Guinea. Mar. Environ. Res..

[88] Pratchett M.S. (2010). Changes in coral assemblages during an outbreak of *Acanthaster planci* at Lizard Island, northern Great Barrier Reef (1995–1999). Coral Reefs.

[89] Jones M.C., Cheung W.W.L. (2015). Multi-model ensemble projections of climate change effects on global marine biodiversity. ICES J. Mar. Sci..

[90] Randall C.J., Szmant A.M. (2009). Elevated Temperature Affects Development, Survivorship, and Settlement of the Elkhorn Coral, Acropora palmata (Lamarck 1816). Biol. Bull..

[91] Foster T., Gilmour J.P., Chua C.M., Falter J.L., McCulloch M.T. (2015). Effect of ocean warming and acidification on the early life stages of subtropical *Acropora spicifera*. Coral Reefs.

[92] Pratchett M.S., Caballes C.F., Cvitanovic C., Raymundo M.L., Babcock R.C., Bonin M.C., Bozec Y.-M., Burn D., Byrne M., Castro-Sanguino C. (2021). Knowledge Gaps in the Biology, Ecology, and Management of the Pacific Crown-of-Thorns Sea Star *Acanthaster* sp. On Australia’s Great Barrier Reef. Biol. Bull..

[93] Deaker D.J., Byrne M. (2022). Crown of thorns starfish life-history traits contribute to outbreaks, a continuing concern for coral reefs. Emerg. Top. Life Sci..

[94] Kayal M., Vercelloni J., Lison de Loma T., Bosserelle P., Chancerelle Y., Geoffroy S., Stievenart C., Michonneau F., Penin L., Planes S. (2012). Predator Crown-of-Thorns Starfish (*Acanthaster planci*) Outbreak, Mass Mortality of Corals, and Cascading Effects on Reef Fish and Benthic Communities. PLoS ONE.

[95] Uthicke S., Logan M., Liddy M., Francis D., Hardy N., Lamare M. (2015). Climate change as an unexpected co-factor promoting coral eating seastar (*Acanthaster planci*) outbreaks. Sci. Rep..

[96] Chivers D.P., McCormick M.I., Allan B.J.M., Ferrari M.C.O. (2016). Risk assessment and predator learning in a changing world: Understanding the impacts of coral reef degradation. Sci. Rep..

[97] Harvey B.J., Nash K.L., Blanchard J.L., Edwards D.P. (2018). Ecosystem-based management of coral reefs under climate change. Ecol. Evol..

[98] Renzi J.J., Shaver E.C., Burkepile D.E., Silliman B.R. (2022). The role of predators in coral disease dynamics. Coral Reefs.

[99] Lourey M.J., Ryan D.A.J., Miller I.R. (2000). Rates of decline and recovery of coral cover on reefs impacted by, recovering from and unaffected by crown-of-thorns starfish *Acanthaster planci*: A regional perspective of the Great Barrier Reef. Mar. Ecol. Prog. Ser..

[100] Jackson J., Donovan M., Cramer K., Lam V. (2014). Status and Trends of Caribbean Coral Reefs: 1970–2012.

[101] Hughes T.P., Barnes M.L., Bellwood D.R., Cinner J.E., Cumming G.S., Jackson J.B.C., Kleypas J., van de Leemput I.A., Lough J.M., Morrison T.H. (2017). Coral reefs in the Anthropocene. Nature.

[102] Hoegh-Guldberg O., Skirving W., Dove S.G., Spady B.L., Norrie A., Geiger E.F., Liu G., De La Cour J.L., Manzello D.P. (2023). Coral reefs in peril in a record-breaking year. Science.

[103] Haywood M.D.E., Thomson D.P., Babcock R.C., Pillans R.D., Keesing J.K., Miller M., Rochester W.A., Donovan A., Evans R.D., Shedrawi G. (2019). Crown-of-thorns starfish impede the recovery potential of coral reefs following bleaching. Mar. Biol..

[104] Cowan Z.-L., Dworjanyn S.A., Caballes C.F., Pratchett M. (2016). Benthic Predators Influence Microhabitat Preferences and Settlement Success of Crown-of-Thorns Starfish (*Acanthaster* cf. Solaris). Diversity.

[105] Kroon F.J., Lefèvre C.D., Doyle J.R., Patel F., Milton G., Severati A., Kenway M., Johansson C.L., Schnebert S., Thomas-Hall P. (2020). DNA-based identification of predators of the corallivorous Crown-of-Thorns Starfish (*Acanthaster* cf. Solaris) from fish faeces and gut contents. Sci. Rep..

[106] Caballes C.F., Pratchett M.S., Raymundo M.L., Rivera-Posada J.A. (2017). Environmental Tipping Points for Sperm Motility, Fertilization, and Embryonic Development in the Crown-of-Thorns Starfish. Diversity.

[107] Pratchett M.S., Cowan Z.-L., Nadler L.E., Caballes C.F., Hoey A.S., Messmer V., Fletcher C.S., Westcott D.A., Ling S.D. (2017). Body size and substrate type modulate movement by the western Pacific crown-of-thorns starfish, *Acanthaster* solaris. PLoS ONE.

[108] Uthicke S., Pratchett M.S., Messmer V., Harrison H. (2021). Limited genetic signal from potential cloning and selfing within wild populations of coral-eating crown-of-thorns seastars (*Acanthaster* cf. Solaris). Coral Reefs.

