# Supplementary material for: Distribution Shifts of Acanthaster solaris Under Climate Change and the Impact on Coral Reef Habitats
Source: Animals (Basel). 2025 Mar 17;15(6):858. doi: 10.3390/ani15060858 (PMC11939250; doi:10.3390/ani15060858)
Supplement: Supplementary file 1 [file animals-15-00858-s001.zip › animals-3512169-supplementary.pdf]

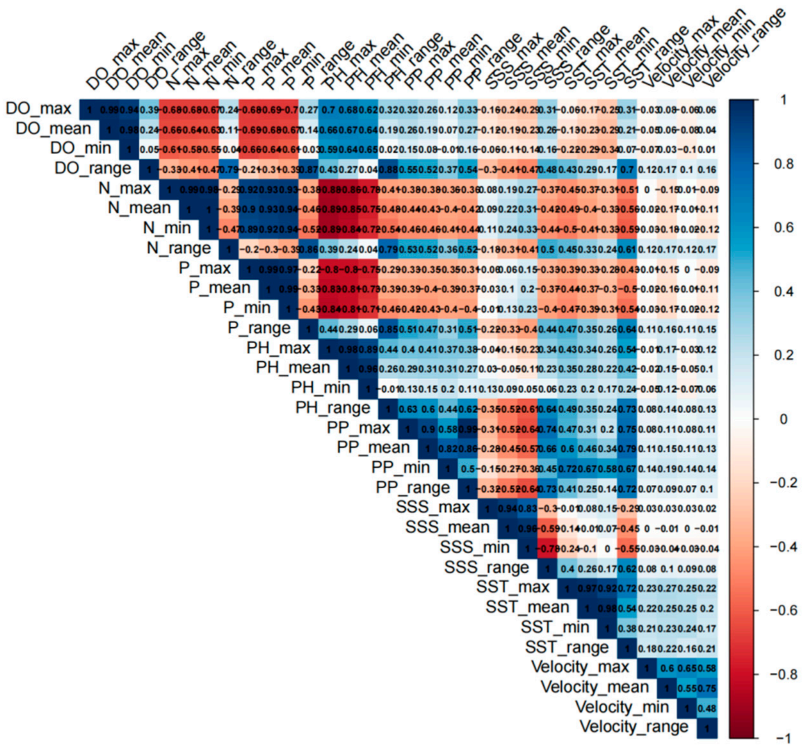

Figure S1. Results of the correlation analysis of variables

Table S1. Alternative abiotic factors

Variable	Acronyms	Unit	Source
Maximum dissolved oxygen	DO_max	mmol/m^3	
Mean dissolved oxygen	DO_mean	mmol/m^3	
Minimum dissolved oxygen	DO_min	mmol/m^3	
The range of dissolved oxygen	DO_range	mmol/m^3	
Maximum nitrate	N_max	mmol/m^3	
Mean nitrate	N_mean	mmol/m^3	
Minimum nitrate	N_min	mmol/m^3	
The range of nitrate	N_range	mmol/m^3	
Maximum phosphate	P_max	mmol/m^3	
Mean phosphate	P_mean	mmol/m^3	
Minimum phosphate	P_min	mmol/m^3	
The range of phosphate	P_range	mmol/m^3	
Maximum pH	PH_max	-	
Mean pH	PH_mean	-	
Minimum pH	PH_min	-	
The range pH	PH_range	-	Bio-ORACLE v3.0 (https://www.bio-oracle.org/) (Assis et al., 2024)
Maxmum primary productivity	PP_max	mmol/m^3	
Mean primary productivity	PP_mean	mmol/m^3	
Minimum primary productivity	PP_min	mmol/m^3	
The range of primary productivity	PP_range	mmol/m^3	
Maximum salinity	S_max	mmol/m^3	
Mean salinity	S_mean	mmol/m^3	
Minimum salinity	S_min	mmol/m^3	
The range of salinity	S_range	mmol/m^3	
Maximum temperature	T_max	ºC	
Mean temperature	T_mean	ºC	
Minimum temperature	T_min	ºC	
The range of temperature	T_range	ºC	
Maximum current velocity	Velocity_max	m/s	
Mean current velocity	Velocity_mean	m/s	
Minimum current velocity	Velocity_min	m/s	
The range of current velocity	Velocity_range	m/s	

Table S2. ODMAP (Overview, Data, Model, Assessment, Prediction) protocol.

ODMAP Section/ Subsection	ODMAP Elements
**OVERVIEW**	
Authorship	▪ **Authors:** Shangke Su, Jinquan Liu, Bin Chen, Wei Wang, Jiaguang Xiao, Yuan Li, Jianguo Du, Jianhua Kang, Wenjia Hu, Junpeng Zhang<br>▪ **Contact e-mail:** sushangke@tio.org.com, huwenjia@tio.org.com<br>▪ **Title:** Climate Change-Driven Distributional Changes of *Acanthaster solaris* and Its Impact on Coral Reef Habitats
Model objective	▪ **Objective:** Ecological inference /explanation; Forecast / transfer<br>▪ **Target outputs:** Habitat suitability maps, environmental variables and species binary maps.
Taxon	*Acanthaster solaris* and *Acropora* coral reef
Location	Region coastal ocean
Scale of analysis	▪ **Spatial extent (Lon/Lat):** 98.28° E-134.97° W; 45.91° S-30.46° N<br>▪ **Spatial Resolution:** 5 arcmin (~10Km)<br>▪ **Temporal extent/time period:** 2000 – 2010, 2090 – 2100(climate scenario SSP1-26, SSP2-4.5, SSP5-85)<br>▪ **Type of extent boundary:** 98.28 ° E-134.97 ° W; 45.91 ° S-30.46 ° N(Ecopolitical: Marine Ecoregions of the World [Spalding et al. 2007])
Biodiversity data overview	▪ **Observation type:** Database data (Obis; Gbif; iNaturalist; Atlas of Living Australia), human survey data, machine observations<br>▪ **Response/Data type:** Presence-only
Type of predictors	Climatic and topographic variables
Conceptual model / hypothesis	▪ **Hypothesis about species-environment relationships:**
Assumptions	▪ Species' distribution is at equilibrium with their environment<br>▪ Species presence data are a representative sample of the species distribution across the study area<br>▪ Pseudo absence data/background data can be treated as absence data

ODMAP Section/ Subsection	ODMAP Elements
	▪ All the key predictor variables of the species under consideration are accounted for in the model
SDM algorithms	**Algorithms:**<br>Maximum Entropy (MAXENT)<br>▪ **Model complexity:** We optimized model complexity via ENMeval R packageA<br>▪ **Model averaging:** N/A
Model workflow	▪ We collected distribution data from online databases as well as field surveys.<br>▪ Reduced sampling bias by keeping one record per 5 arcmin grid cell.<br>▪ Selected marine predictors based on their ecological relevance and collinearity.<br>▪ Fitted SDMs using MaxEnt algorithm, optimized model parameters via ENMeval R package.<br>▪ Assessed model performance using five-fold cross-validation approach.<br>▪ predicted starfish and coral distribution under present (2000-2010) and future (2090 to 2100) scenarios. removal of the least contributing variables(climate scenario SSP1-26, SSP2-4.5, SSP5-85).
Software, codes, and data	▪ **Modelling platform:** Maxent software; We performed all the analyses in R version 4.1.0, mainly using ENMeval, raster, spocc, sf R packages.<br>▪ **Code:** Code will be available upon request<br>▪ **Data:** We first collected all occurrence records of A. planci from four databases: Global Biodiversity Information Facility (https://www.gbif.org/); Ocean Biodiversity Information System (https://obis.org/); iNaturalist (https://www.inaturalist.org/); and Atlas of Living Australia (https://www.ala.org.au/).These records were then spatially filtered according to the A.solaris distribution ecoregions as defined by Uthicke et al.,

ODMAP Section/ Subsection	ODMAP Elements
**DATA**	
Biodiversity data	▪ **Taxon names:** *Acanthaster solaris and Acropora coral reef*<br>▪ **Taxonomic reference system:** N/A<br>▪ **Ecological level:** Species level<br>▪ **Data source:**<br>Species presence data were derived from the global database(Obis; Gbif; iNaturalist; Atlas of Living Australia)<br>▪ **Sampling design:** N/A<br>▪ **Sample size:** 11,483 occurrence points for *A. solaris*; 289,440 global occurrence records for *Acropora* species<br>▪ **Absence data:** N/A<br>▪ **Data cleaning and filtering:** We cleaned distribution data by removing records outside species known natural range and keeping one record per 5 arcmin grid cell.
Data partitioning	▪ Created 2,000 km buffer around species occurrence records, then selected 10,000 random points as background data.
Predictor variables	▪ **Predictor variables:**<br>Based on the collinearity results and ecological relevance, we ultimately selected 12 environmental variables, including depth, DO_mean, and KDPAR_mean (Table 1)<br>▪ **Data sources:**<br>1. Environmental variables: Bio-ORACLE (Tyberghein et al. 2012; Assis et al. 2017)<br>2. Bathymetry: GEBCO(gebco.net)<br>3. Distance from coral reef: SDM outputs<br>▪ **Data processing:** The raster layers were extracted, projected, and resampled using Arcmap10.2.1<br>▪ **Spatial resolution of raw data:** 5 arcmin, 5 arcmin<br>▪ **Projection:** WGS84

ODMAP Section/ Subsection	ODMAP Elements
**MODEL**	
Variable pre-selection	▪ We quantified the correlation levels between abiotic predictors via the Pearson's correlation coefficient (r) and excluded strongly collinear variables (i.e., |r| > 0.7) to reduce their possible effects on model fitting (Dormann et al. 2013).
Multicollinearity	▪ Multicollinearity among the predictor variables were tested using the ENMeval package in R.
Model settings	▪ We optimized model parameters via ENMeval R package
Model estimates	▪ **Model coefficient:** TSS, KAPPA<br>▪ **Variable importance:** Importance of predictor variables in the three different models were calculated
Model averaging / ensembles	▪ N/A
Non-independence	▪ No test was performed to test for non-independence of the models.
**ASSESSMENT**	
Performance statistics	▪ **Performance statistics estimated on training data:** N/A
Plausibility checks	▪ **Response plots:** Ecological plausibility was tested using the response curves for the predictor variables.
**PREDICTION**	
Prediction output	▪ N/A
Uncertainty quantification	▪ **Algorithmic uncertainty:** N/A<br>▪ **Reality check:** Evaluation of the results based on database data and data from the literature published over the years, and accuracy testing in conjunction with survey data
